# Notes and News

**Published:** 1897-11-20

**Authors:** 


### NOTES AND NEWS.

(Collected and Communicated.)

Sir Henry Burdett has been elected a Vice-President of the Chichester Infirmary for the ensuing year.

The exhibition of the Queen's Jubilee presents at the Imperial Institute has already produced £500 for the Prince of Wales's Fund.

It is stated that four of the large City companies have combined to open and endow a ward of thirty beds at St. Thomas's Hospital as a Jubilee memorial. This will entail an expenditure of several thousands.

The proposal to acquire the disused Naas Prison to give extra accommodation to the Richmond Lunatic Asylum, Dublin, has been abandoned as too expensive and the building being too far from Dublin. The asylum has now the use of the Female Penitentiary at Grange Gorman, which it has acquired.

The memory of two naval surgeons is to be perpetuated at Haslar by the erection of a memorial tablet in the hospital. Surgeon Charles Fyfe and Surgeon Richard Way both died in the execution of their duty during the Benin expedition, and the tablet is erected to their memory by brother medical officers.

A terrible story of the condition of the large body of army reservists comes from France. Skin epidemics constantly appear amongst them, and these are traced to the clothing with which they are supplied, and which is described as frequently filthy and tattered in condition. When it is considered that the reserve force numbers about 100,000, the extent of the possible evil may be estimated.

Mr. C. Hawkins' Jubilee gift to the Essex and Colchester Hospital is now completed. It consists of a much-needed porter's lodge, erected at the north-east entrance of the hospital. It is expected that it will remove one of the difficulties of the institution, which has been to retain the services of a porter, one dozen men having been employed in succession during ten years.

Dr. Walter S. A. Griffith has been elected president of the Medical Defence Union, in the place of Mr. Victor Horsley, who has joined the General Medical Council.

The number of typhoid cases at Maidstone continues to decline, and a large number of convalescents are at Sandgate recruiting their health. At Lynn the number of cases exceeds 390; the deaths number upwards of 30.

The Scarborough Hospital is not forgotten by the generous, and it has recently received two interesting presents from Miss Emilie Ornstein. One is a complete Röntgen-ray apparatus, and the other a baby incubator.

The plague continues to advance in Poona, as many as 1,100 victims being reported weekly. At Calcutta preventive measures are being prosecuted with vigour. At Madras a few cases have been introduced from Poona, but those attacked have been completely segregated.

The recent outbreak of typhoid fever, which is now abating, at Clifton is traced to one source, namely, to the milk supply coming from one particular farm, by which passes a stream contaminated with sewage. The water from the stream was used to "cleanse" the dairy utensils.

Lieutenant Costello, who so distinguished himself at the commencement of the present Indian campaign by risking his life to save a comrade, is son of a retired army doctor, Surgeon-Colonel Costello, who also distinguished himself in service. The Queen has conferred the Victoria Cross upon Lieutenant Costello.

A very interesting manuscript has been found in the library of the Vatican. This is a set of prescriptions used in the treatment of the eye disease from which Michael Angelo suffered. The directions and advice of the various doctors have been preserved, and all have been published in Italian by Dr. Berger, who discovered the manuscript.

Lady Dickson Poynder has laid the foundation-stone of a cottage hospital at Chippenham. It will contain one male and one female ward with three beds in each, two single wards, and nurses and servants' quarters. The cost has been estimated at £1,400, of which £500 has been subscribed by Sir J. Dickson Poynder, and the rest promised from other sources, besides sufficient funds for the first year's maintenance.

A new wing has been added to the Ingham Infirmary, North Shields. It is to be called the John Readhead Wing. There are two large wards for 20 beds, and four small ones with two beds in each. Convalescent, day, and dining rooms, together with offices and day accommodation for nurses, are also provided. The cost of building is estimated at £5,928, and of furnishing at £1,500.

Extensive alterations and additions have been made at the Ulster Eye, Ear, and Throat Hospital, and pains have been taken to bring it up to modern requirements. The operating theatre has been placed at the top of the building, so that adequate light has been easily secured. Eight new private wards have been added, and also new day rooms for patients of both sexes.

